# Supplementary material for: Synthetic mammalian transgene negative autoregulation
Source: Mol Syst Biol. 2013 Jun 4;9:670. doi: 10.1038/msb.2013.27 (PMC3964311; doi:10.1038/msb.2013.27)
Supplement: Supplementary Information — Supplementary figures S1-14, Supplementary tables S1-S2 [file msb201327-s1.pdf]

Supplementary Material

Supplementary Figures

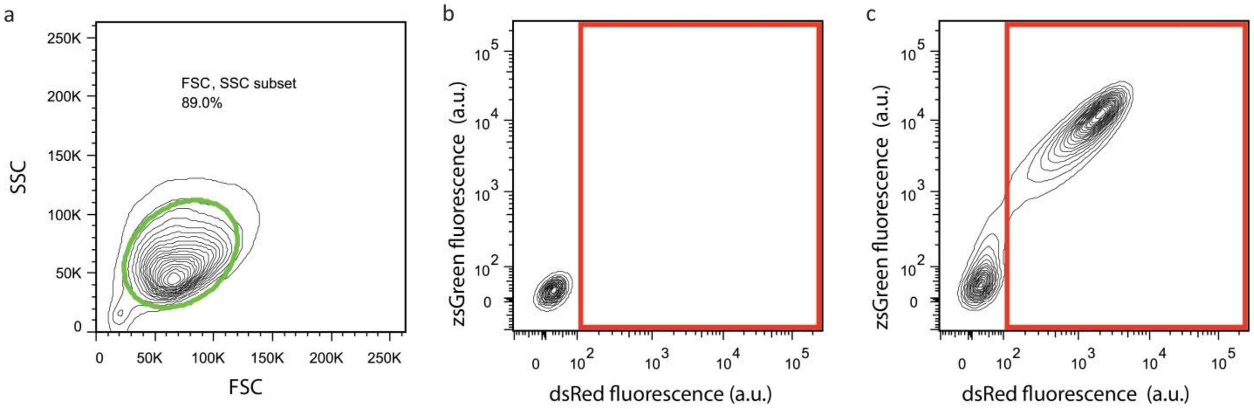

Supplementary Figure1. Flow cytometry data gating and processing. (a) A gate based on the forward and side scatter is first used to select single cell events. **(b)** The gated population is projected for dsRed and zsGreen. The particular case is a population of H293 cells without any fluorescence. Subsequently, we gated the constitutive protein positive events (e.g. dsRed for the negative feedback) at the threshold of negative cells**. (c)** The particular case is a population is the negative feedback. ■

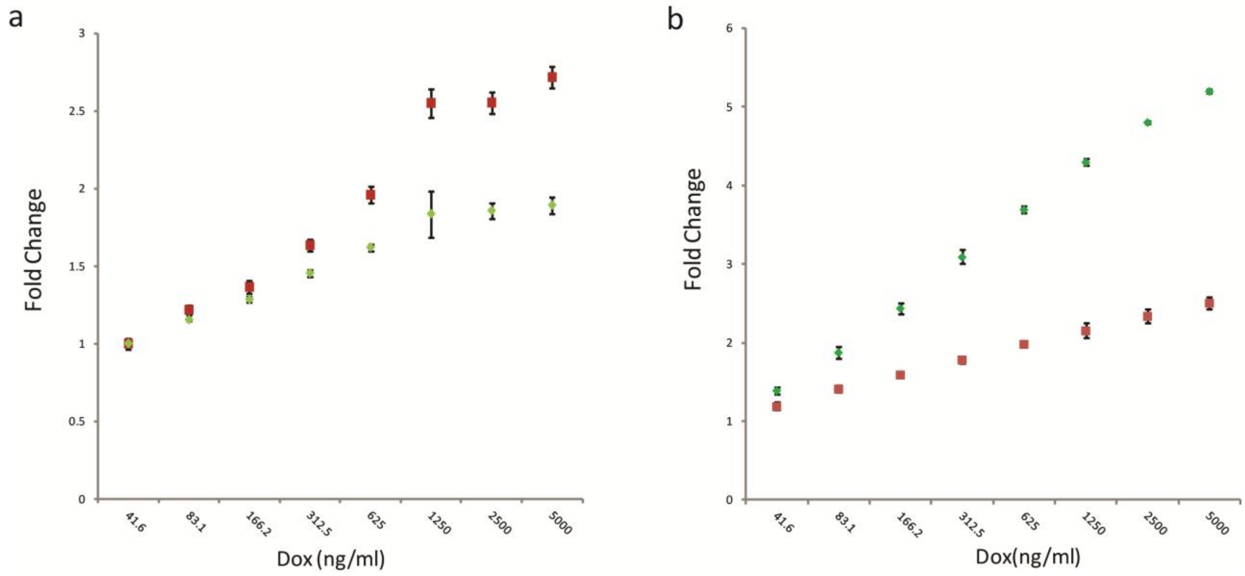

Supplementary Figure 2. Doxycycline titrations for the negative feedback loop and the control architecture. The zsGreen protein in green diamonds and the dsRed in red squares. Error bars show the standard deviation of triplicate experiments. **(a)** Absolute change of mean fluorescent levels of dsRed and zsGreen for the NFL. **(b)** Absolute change of mean fluorescent levels of dsRed and zsGreen for the control architecture.■

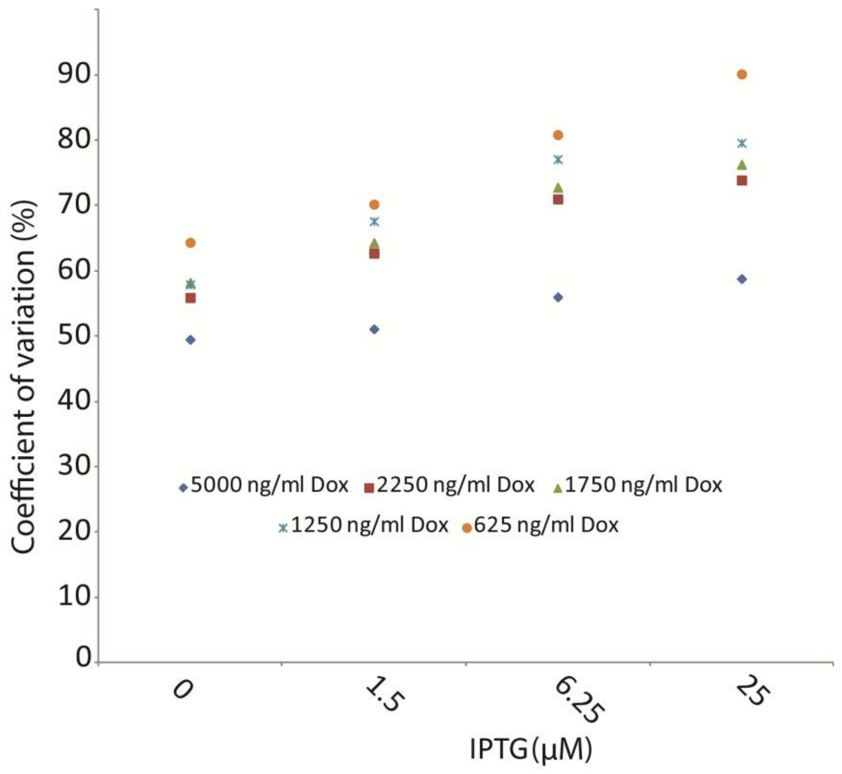

Supplementary Figure 3. Coefficient of variation versus IPTG concentration for the negative feedback loop for various concentrations of doxycycline. ■

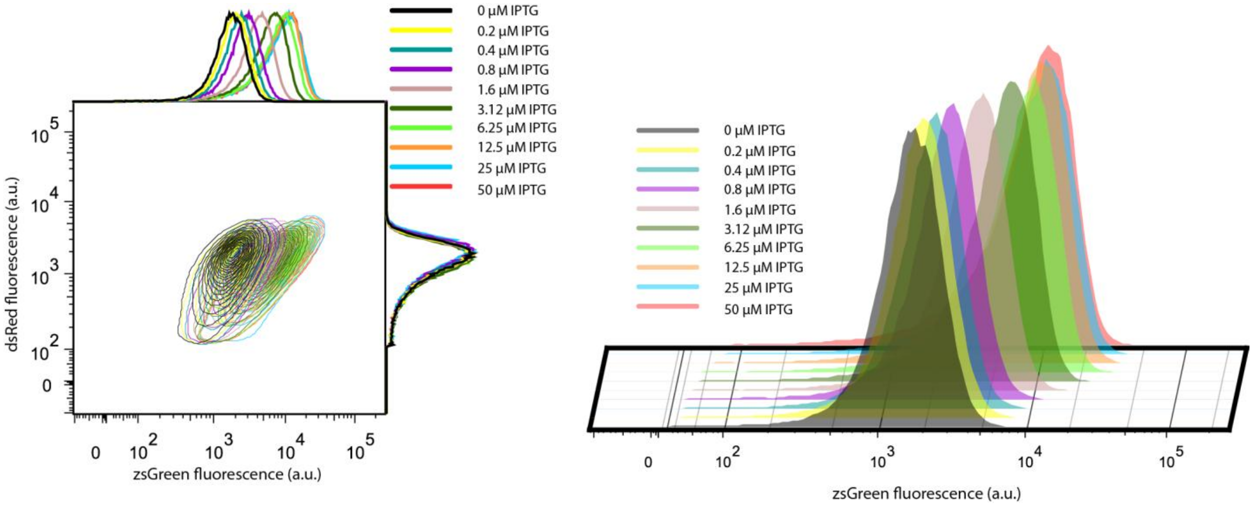

Supplementary Figure 4. IPTG Titrations for the negative feedback loop. The high Doxycycline case. DsRed positive cells are illustrated and the corresponding histograms for each output are also presented. ■

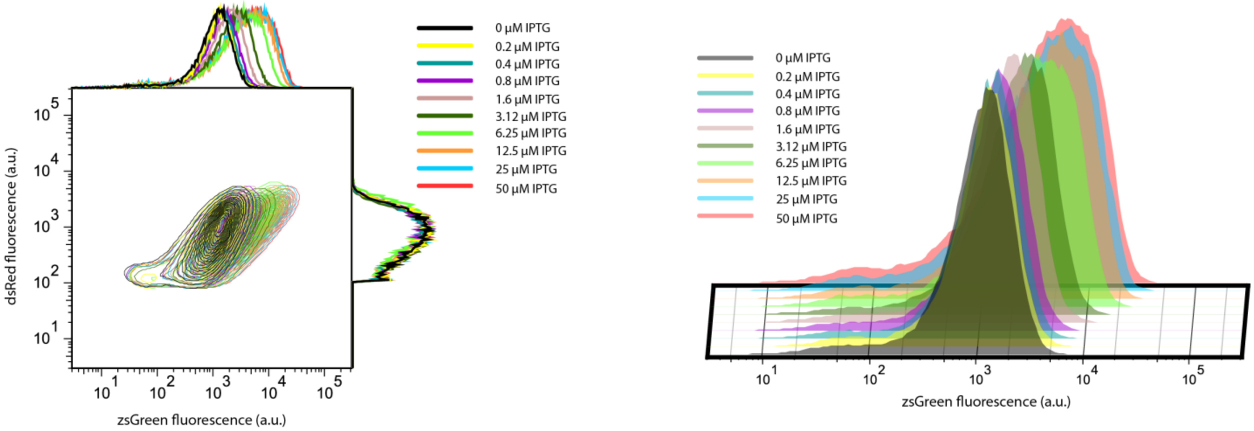

Supplementary Figure 5. IPTG Titrations for the negative feedback loop. The low Doxycycline case. DsRed positive cells are illustrated and the corresponding histograms for each output are also presented.

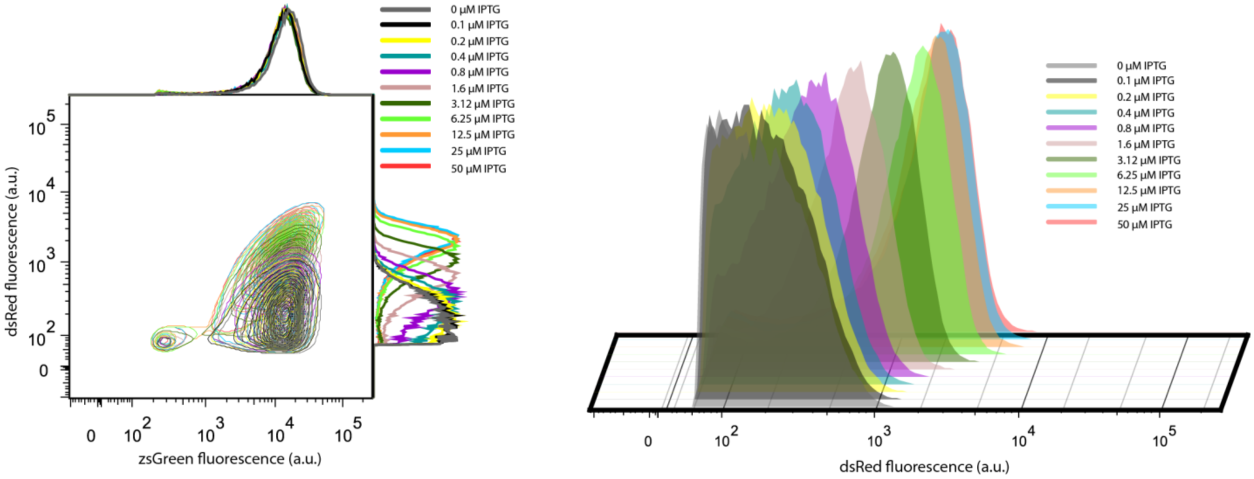

Supplementary Figure 6. IPTG Titrations for the cascade. The high Doxycycline case. DsRed positive cells are illustrated and the corresponding histograms for each output are also presented. ■

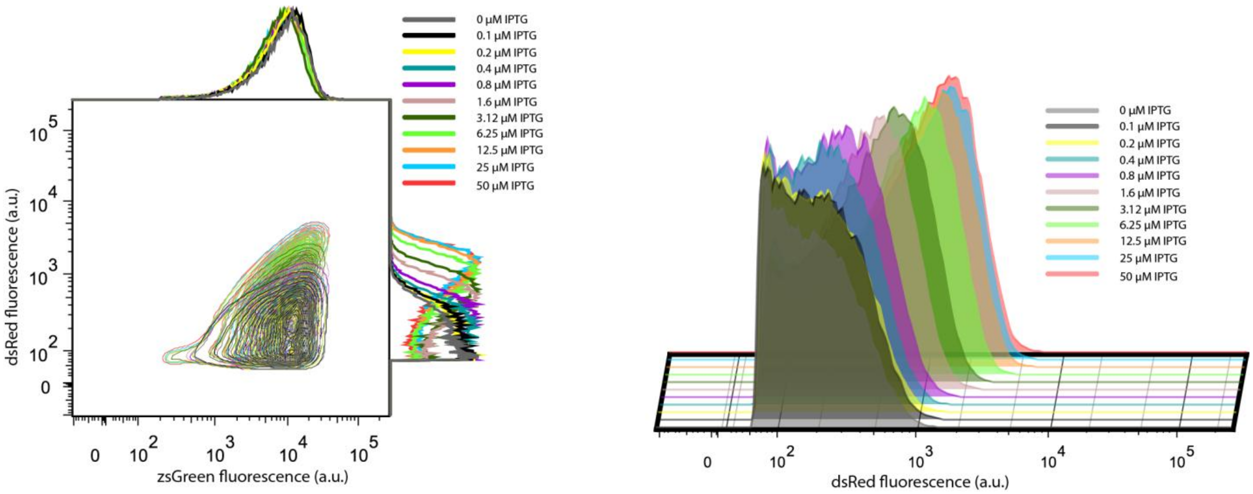

Supplementary Figure 7. IPTG Titrations for the cascade. The low Doxycycline case. DsRed positive cells are illustrated and the corresponding histograms for each output are also presented. ■

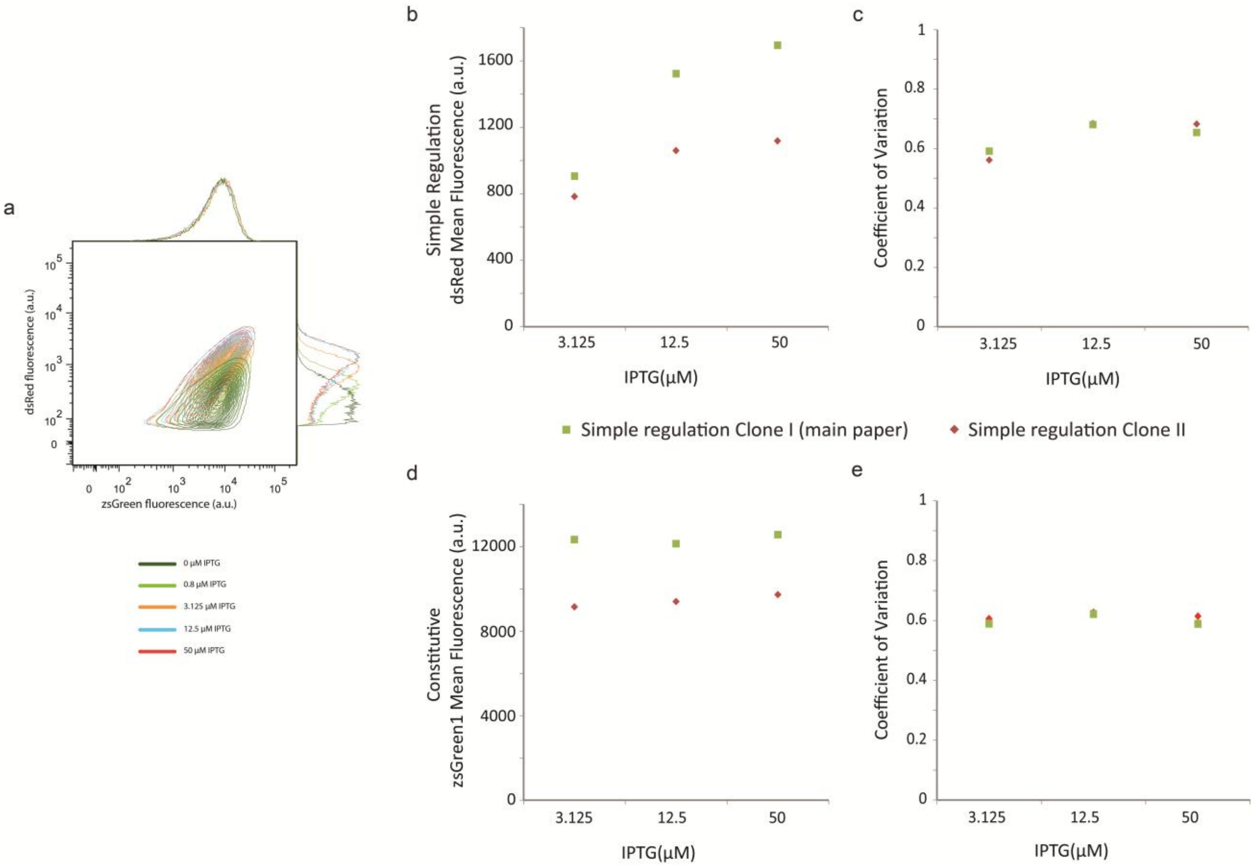

Supplementary Figure 8. Comparison of mean fluorescence and coefficient of variation between the main paper simple regulation clone and another simple regulation transgene.■

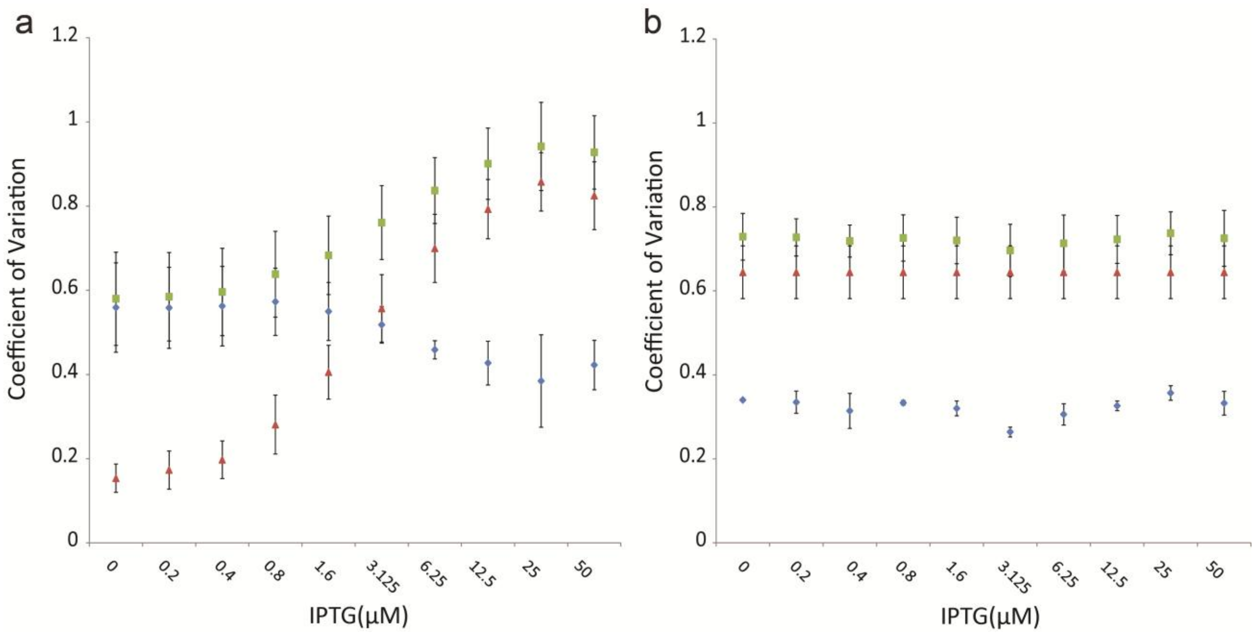

Supplementary Figure 9. Coefficient of variation for the control architecture. Local, global and total noise in gene expression of the control architecture. **(a)** Coefficient of variation of dsRed protein for low DOX, **(b)** Coefficient of variation of zsGreen protein for low DOX.■

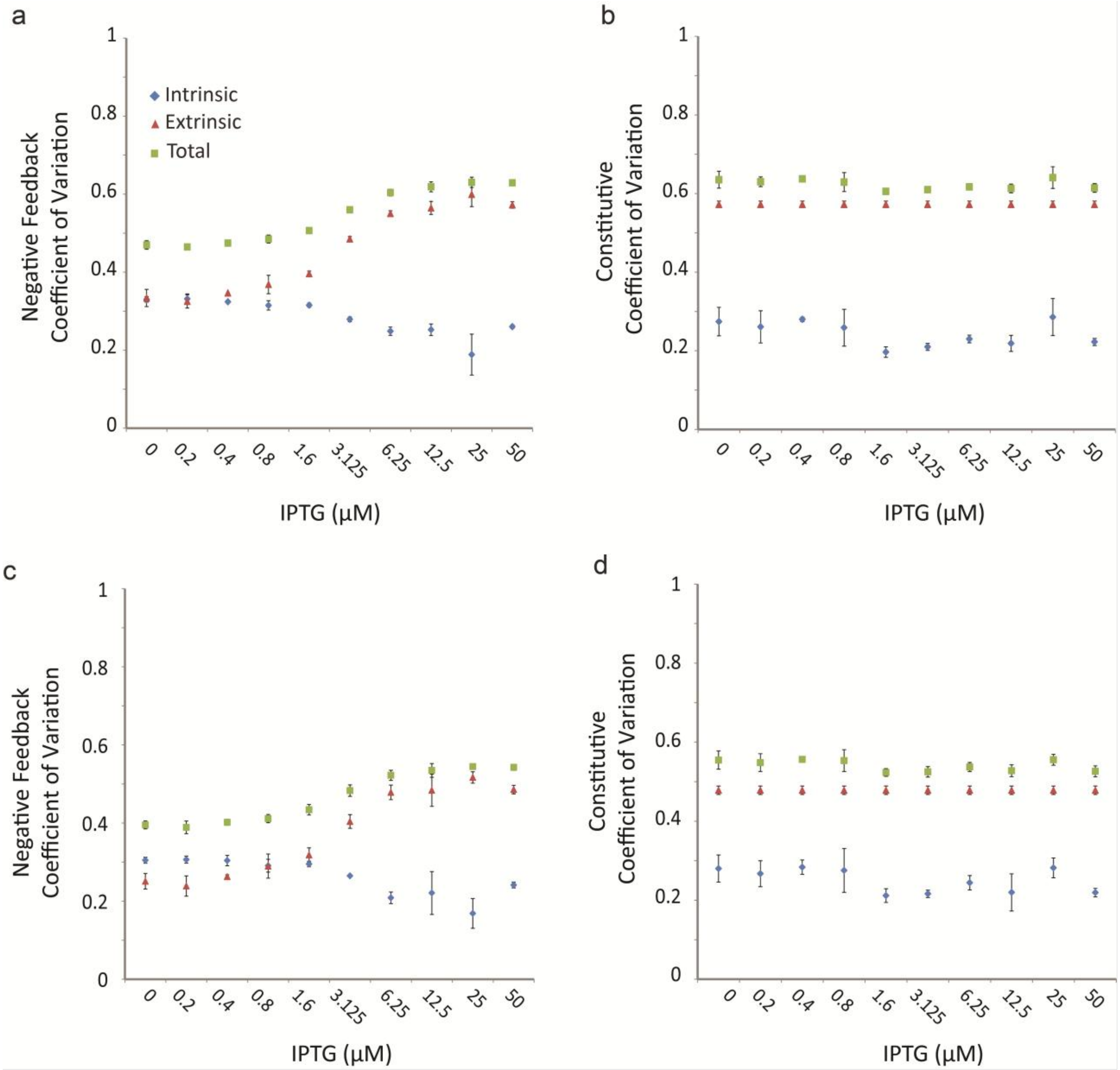

Supplementary Figure 10. Effect of forward scattering vs. side scatter gate on the extrinsic noise. The top panels (a and b) correspond to the original gate (SSC ~20k-120k and FSC ~20k-120k gate, supplementary figure 1) while panels c and d were prepared using smaller gate (SSC ~40k-50k and FSC ~70k-80k gate).■

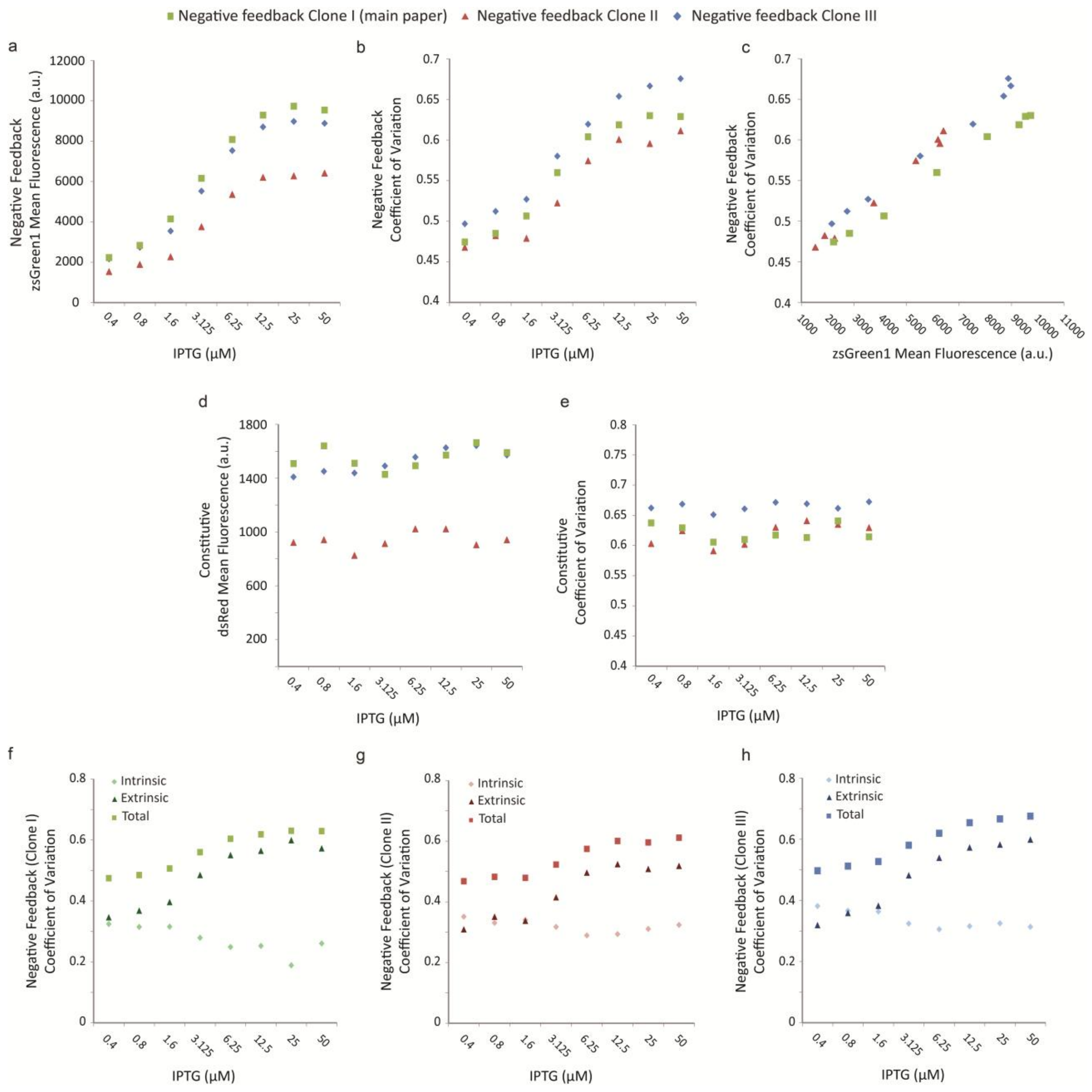

Supplementary Figure 11. Comparison of mean fluorescence and coefficient of variation between the main paper negative feedback clone and two different negative feedback transgenes.■

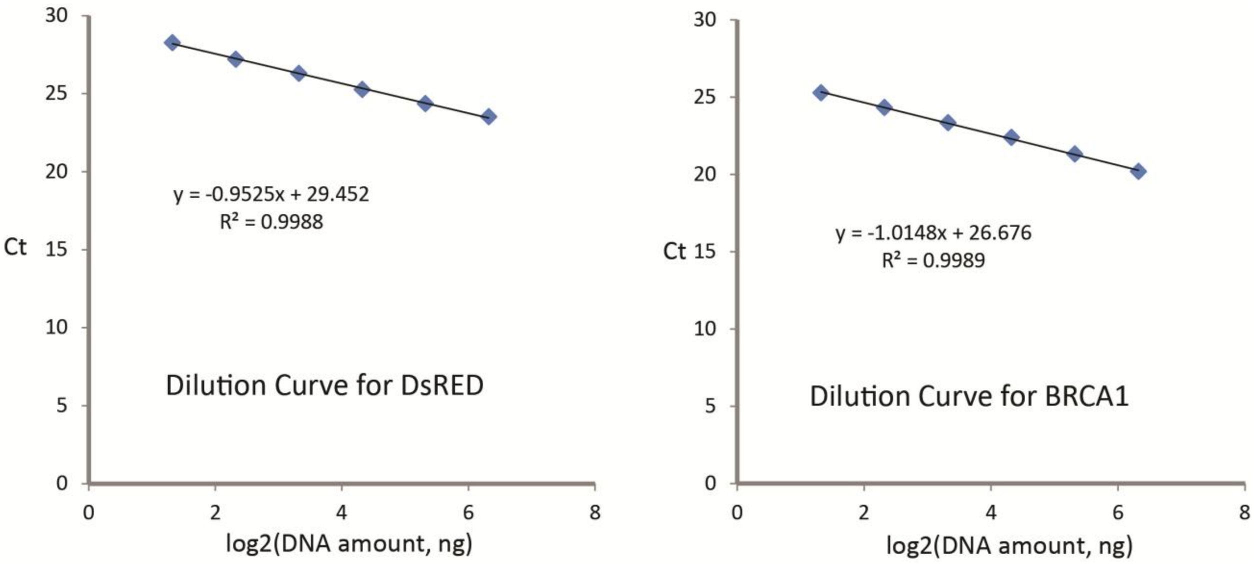

Supplementary Figure 12. Determination of PCR amplification efficiencies for DsRED and BRCA1 gene targets. The dilution curves (DsRED and BRCA1) were plotted as $\log_2$(DNA amount, ng) versus Ct. The PCR amplification efficiency E was calculated as: $2^{(-1/\text{slope of the dilution curve})}$-1. E_{DsRED} was determined as 1.07, and E_{BRCA1} as 0.98.■

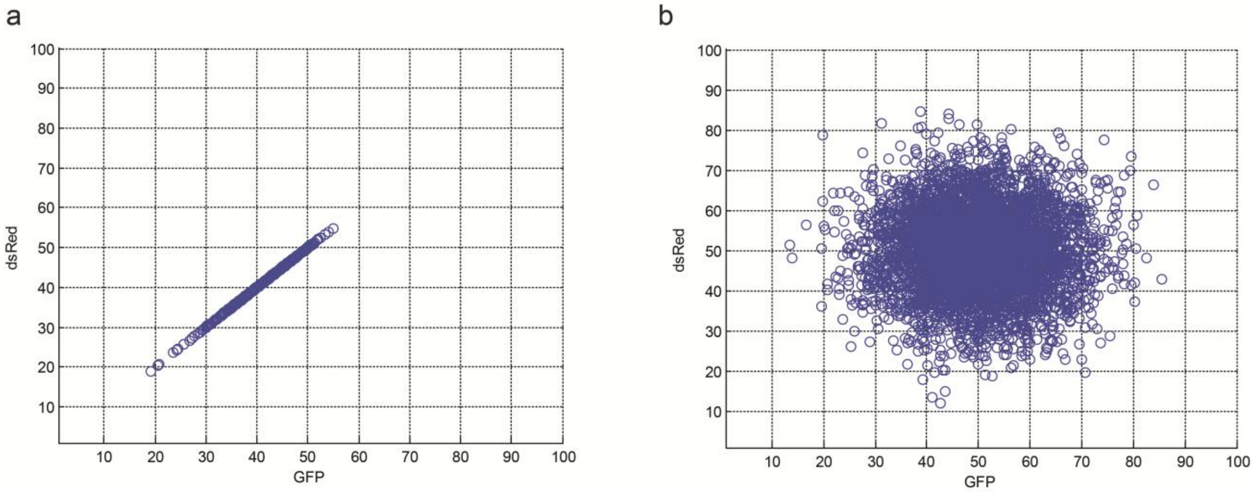

Supplementary Figure 13. Simulations where the intrinsic and extrinsic noise change separately. **(a)** We vary the strength of transcription of a single bidirectional promoter coding for two fluorescent proteins,

leading to perfectly correlated fluorescence quantities. **(b)** We vary the strength of transcription of two fluorescent genes independently.■

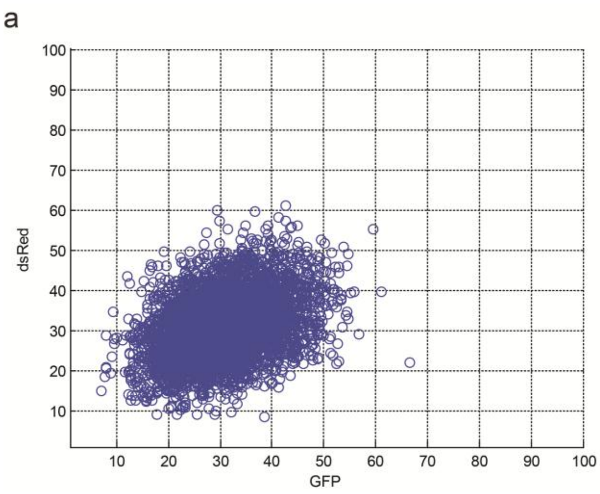

Supplementary Figure 14. Simulations where the intrinsic and extrinsic noise change simultaneously.■

Transgene copy number

Real-time quantitative PCR has been used as an alternative to Southern blot or fluorescence in situ hybridization for detection of gene copy numbers[1]. Various studies demonstrated that this method is accurate enough compared to Southern blot. For example, in Table 2 from "Determination of Cytochrome P450 2D6 (CYP2D6) gene copy number by real-time quantitative PCR"[2], the estimations of CYP2D6 gene copies from real-time quantitative PCR match with those from Southern blotting. The average copy numbers of DsRED of all stable clones were estimated by the delta delta Ct method as follows: $2^{-\Delta\Delta Ct} = ((1 + E_{DsRED})^{-\Delta Ct,DsRED}) / ((1+E_{BRCA1})^{-\Delta Ct,BRCA1})$, where E_{DsRED} is the PCR amplification efficiency for DsRED and E_{BRCA1} for BRCA1 (endogenous reference gene)[3].

The PCR primers are: DsRED forward primer: 5'- ctccaccacggtgtagtcct-3'; DsRED reverse primer: 5'- agaccgtgtacaaggccaag-3'; BRCA1 forward primer: 5'- gagcgtcccctcacaaataa-3'; and BRCA1 reverse primer: 5'- tgctccgtttggttagttcc-3'. The control stable HEK293 cell line was generated by Flp-In system (Invitrogen) and contains one copy of DsRED transgene[4]. All genomic DNA samples were extracted using DNeasy Blood and Tissue kit (Qiagen). To determine the PCR amplification efficiency, genomic DNAs from the control cell line were used to generate the dilution curve of $\log_2$(DNA amount, ng) vs. Ct. E_{DsRED} was calculated as 1.07, and E_{BRCA1} as 0.98. The PCR conditions were as: 95 degree for 3 minutes, followed by 40 cycles of 95 degree for 15 seconds and 60 degree for 30 seconds. For each stable clone, triplicates (50 ng of genomic DNA) were performed and the average copy numbers were calculated as the mean ± SD. For statistical analysis, z scores were calculated against estimated integer copy numbers, and $-1.96<z<1.96$ was determined as no statistical difference (corresponding to 95% confidence interval).

Determination of PCR amplification efficiency: 80 ng, 40 ng, 20 ng, 10 ng, 5 ng and 2.5 ng of genomic DNAs were extracted from the control cell line using DNeasy Blood and Tissue kit (Qiagen). The PCR primers are: DsRED forward primer: 5'- ctccaccacggtgtagtcct-3'; DsRED reverse primer: 5'-

agaccgtgtacaaggccaag-3'; BRCA1 forward primer: 5'- gagcgtcccctcacaaataa-3'; and BRCA1 reverse primer: 5'- tgctccgtttggttagttcc-3'. The PCR conditions were as: 95 degree for 3 minutes, followed by 40 cycles of 95 degree for 15 seconds and 60 degree for 30 seconds. The dilution curves were plotted (**Supplement Fig. 12**) as $\log_2$(DNA amount, ng) vs. Ct. The PCR amplification efficiency E was calculated as: $2^{(-1/\text{slope of the dilution curve})}-1$.

Supplementary Table I. Transgene clones and the resulting number of integrations.

Clone	Gene Copy Average	Standard Deviation
**Negative Feedback (NF Clone I: L6.89.14)**	1.026716891	0.205356713
**Simple Negative Regulation (SNR Clone I: V2.42.1)**	1.986616106	0.063272704
**Single integration clone**	1.005016457	0.127722277
**Simple Negative Regulation (SNR Clone II: V2.52.2)**	1.024331368	0.09450017

Supplementary Table II: The values for alpha obtained for the manuscript clones.

IPTG (µM)	Negative Feedback High Dox	Negative Feedback Low Dox	Simple Negative Regulation High Dox	Simple Negative Regulation Low Dox
**50**	1	1.22	1.11	1.28
**25**	1.046293	1.280207	1.170337	1.332335
**12.5**	0.986329	1.211581	1.232468	1.230991
**6.25**	0.961434	1.195583	1.064725	1.084425
**3.125**	0.847654	1.111019	0.868676	0.861535
**1.6**	0.692237	0.864742	0.684977	0.626666
**0.8**	0.64337	0.749677	0.548974	0.431871
**0.4**	0.605457	0.677382	0.389802	0.303799
**0.2**	0.568188	0.649937	0.323275	0.265747
**0**	0.5828	0.630114	0.337724	0.23634

Theory

Stochastic events which govern the concentration of a single protein, such as the synthesis and degradation of that protein, are referred to as "intrinsic" or "local" noise. Such random fluctuations can propagate along regulation pathways, with the consequence that protein distributions along a pathway appear correlated[5, 6]. However, even proteins from different regulation pathways show correlation[5, 7]. This arises from stochastic variations in quantities which affect the regulation of all genes[5, 6], such as in polymerase copies or cell cycle phase. As a consequence, a strongly expressing constitutive promoter is expected to have little intrinsic noise, while a weak promoter will have high intrinsic noise[8, 9]. In addition, two identical, independently regulated promoters are expected to have the same extrinsic noise, which arises through global effects[5, 7].

The total noise observed in a fluorescent reporter distribution arises through the combination of these "global" or "extrinsic" fluctuations together with the fluctuations in that protein's local regulation machinery ("intrinsic" noise)[5]. The intrinsic noise and extrinsic noise squared, sum to the CV-squared of the fluorescent reporter[5]. Using this notation, let angle brackets indicate that an average is taken with extrinsic variables held fixed, and let an overbar indicate an average where intrinsic variables are fixed. Then the three noises, intrinsic, extrinsic, and total, can be written in terms of P, the observed distribution of reporter protein:

$$\mathrm{n}_{\mathrm{tot}}^2 = \frac{\overline{\langle P^2 \rangle} - \left(\overline{\langle P \rangle}\right)^2}{\left(\overline{\langle P \rangle}\right)^2}$$

$$\mathrm{n}_{\mathrm{int}}^2 = \frac{\overline{\langle P^2 \rangle - \langle P \rangle^2}}{\left(\overline{\langle P \rangle}\right)^2}$$

$$\mathrm{n}_{\mathrm{ext}}^2 = \frac{\overline{\langle P \rangle^2} - \left(\overline{\langle P \rangle}\right)^2}{\left(\overline{\langle P \rangle}\right)^2}$$

$$\mathrm{n_{tot}^2 = n_{int}^2 + n_{ext}^2}$$

For intrinsic noise, the authors of[5] take the variance of the intrinsic variables, $\langle P^2\rangle - \langle P\rangle^2$, then estimate the expected value of this variance, denoted by the overbar, and subsequently divide by the mean squared of P. For extrinsic noise, the authors take the expected value of P with respect to intrinsic variables, then the variance of $\langle P\rangle$, and finally divide by the mean squared of P. For the total noise (CV-squared), the variance of P is divided by the mean squared.

Note that with a single reporter, the noises can't be estimated unless both the intrinsic and extrinsic variables are observed. However, in the standard two-reporter experiment, the extrinsic noise becomes the normalized covariance of two reporters that are independently regulated and identically distributed. The reason[5] is that in a single cell, the extrinsic variable is fixed, so the quantity $\overline{\langle P\rangle^2}$ can be calculated as the average product of the two reporters $\overline{\langle P^{(1)}P^{(2)}\rangle}$, then since $\overline{\langle P^{(1)}\rangle} = \overline{\langle P^{(2)}\rangle}$ the extrinsic noise becomes the normalized covariance of the two reporters:

$$n_{ext}^2 = \frac{\overline{\langle P^{(1)}P^{(2)}\rangle} - \left(\overline{\langle P^{(1)}\rangle}\right)\left(\overline{\langle P^{(2)}\rangle}\right)}{\left(\overline{\langle P^{(1)}\rangle}\right)\left(\overline{\langle P^{(2)}\rangle}\right)}$$

and the intrinsic noise becomes the normalized RMS difference from $P^{(1)} = P^{(2)}$, so that the sum of intrinsic and extrinsic is twice the CV of one reporter.

In this paper we examine more complicated regulatory mechanisms where it is not feasible to construct two identically-regulated reporters (or impossible to obtain identical reporter statistics). We define a new formulation and we will obtain the previous results as a special case, where the extrinsic noise is the normalized covariance and the components sum to the total noise.

Let X be the observed reporter protein, and A and B are the intrinsic and extrinsic variables; if we assume a multiplicative model and that the variables are independently distributed, we can derive the following intrinsic/extrinsic noise breakdown:

$$\frac{\mathbf{E}\left((\mathbf{X}-\boldsymbol{\mu}_{\mathbf{X}})^{2}\right)}{\boldsymbol{\mu}_{\mathbf{X}}{}^{2}}=\frac{\mathbf{E}\left((\mathbf{A}-\boldsymbol{\mu}_{\mathbf{A}})^{2}\right)}{\boldsymbol{\mu}_{\mathbf{A}}{}^{2}}+\frac{\mathbf{E}\left((\mathbf{B}-\boldsymbol{\mu}_{\mathbf{B}})^{2}\right)}{\boldsymbol{\mu}_{\mathbf{B}}{}^{2}}$$

Such a multiplicative model can be motivated as follows. Suppose gene X is activated by two factors; one (A) is an intrinsic variable such as a transcription factor, and the other (B) is an extrinsic variable, such as RNA polymerase. Suppose both factors must be present for transcription, in the complex ABX. We have four reaction equations:

$$\mathrm{X}+\mathrm{A}\leftrightarrow\mathrm{AX}$$

$$\mathrm{X}+\mathrm{B}\leftrightarrow\mathrm{BX}$$

$$\mathrm{AX}+\mathrm{B}\leftrightarrow\mathrm{ABX}$$

$$\mathrm{BX}+\mathrm{A}\leftrightarrow\mathrm{ABX}$$

This results in the following algebraic equations at steady-state:

$$\mathrm{k_{r1}[AX]+k_{r2}[BX]=k_{f1}[A][X]+k_{f2}[B][X]}$$

$$\mathrm{k_{f1}[A][X]+k_{r3}[ABX]=k_{r1}[AX]+k_{f3}[B][AX]}$$

$$\mathrm{k_{f2}[B][X]+k_{r4}[ABX]=k_{r2}[BX]+k_{f4}[A][BX]}$$

$$\mathrm{k_{f4}[A][BX]+k_{f3}[B][AX]=k_{r4}[ABX]+k_{r3}[ABX]}$$

For the gene activity, we take the ratio of active complex ABX to total gene copies:

$$\mathrm{X_{active}}=\frac{\mathrm{[ABX]}}{\mathrm{[X]+[AX]+[BX]+[ABX]}}$$

This simplifies to an expression in terms of A and B (we drop most of the constants):

$$\mathrm{X_{active}}=\frac{\mathrm{[A][B]+[A]^{2}[B]+[A][B]^{2}}}{\mathrm{k+[A]+[B]+[A]^{2}+[B]^{2}+[A]^{2}[B]+[A][B]^{2}}}$$

Which, for small, unsaturated concentrations of A and B, looks like:

$$X_{active} \approx \frac{[A][B]}{k}$$

Intuitively, in this multiplicative approximation, a polymerase fluctuation of 10% is expected to change gene activity by 10% (with an unsaturated promoter). Compare this to an additive noise model: now, the same polymerase fluctuation of 1000 molecules is expected to change gene output by 1000 molecules, regardless of whether the output is currently regulated at 10000 molecules or at 100 molecules. Thus the multiplicative model makes physical sense for positive variables, where a reporter with 100 molecules cannot have an uncertainty of 1000 molecules.

We generalize this multiplicative model and assume the observed random variable is a function of its independent component sources (A and B, the intrinsic and extrinsic variables) of the following general form:

$$X = A^a B^b$$

i.e. where a and b are not necessarily both equal to 1. These sensitivity coefficients must appear as powers because multiplied coefficients fall out as a single constant in the next step. It is convenient to convert this to a linear model (for ease of calculation), by taking the logarithm:

$$\log(X) = a \log(A) + b \log(B)$$

For ease of notation, we drop the log functions and just use the original variable names.

$$X = a A + b B$$

Here we need to calculate the contributions of A (intrinsic) and B (extrinsic) to the total observed noise of X. In general, summing two independent random variables A and B with variance $\mathrm{Var}(A)$ and $\mathrm{Var}(B)$ results in the following variance:

$$\mathrm{Var}(aA + bB) = a^2\mathrm{Var}(A) + b^2\mathrm{Var}(B) + 2ab\mathrm{Cov}(A, B) = a^2\mathrm{Var}(A) + b^2\mathrm{Var}(B)$$

The last equality holds because the intrinsic and extrinsic components have been defined to have no covariance term: any fluctuation which affects two identical reporters is an extrinsic variable, and all remaining noise observed is intrinsic. The variances of logarithms returned by this method are approximately the normalized variances (CV-squares) of the original quantities; we discuss this point later.

Elowitz et al.[7] argues that two identically regulated reporters with the same mean and variance should have the same extrinsic noise, and uses this fact to calculate the noise components. We introduce the following modification to extend the intrinsic/extrinsic breakdown to cases where one reporter is not constitutive, and hence may not obey the Elowitz et al. assumptions. For a regulated reporter, noise may propagate along the regulatory pathway, changing the reporter's susceptibility to global fluctuations (supposing that this reporter is in the same cell as a constitutive reporter). We capture this asymmetric effect by adding a sensitivity coefficient to the regulated reporter for a two-reporter noise breakdown.

Let Y be the constitutive reporter and X a regulated reporter (controlled by an inducer, in our case IPTG). A is a function of all extrinsic variables, B and C are the intrinsic variables for each promoter, and α is a coefficient which is 1 for two constitutive promoters with identical reporter statistics (as in Elowitz et al.) but varies depending on the regulation of X. α represents an aggregated susceptibility to fluctuating variables which affect both reporters, similar to the quantity H used in Paulsson[10] to denote the logarithmic gain of an interaction:

$$X = A^{\alpha}B$$

$$Y = AC$$

With α placed as a power of A, we have defined $(\ln(X|B) - \langle\ln(X|B)\rangle)/(\ln(A) - \langle\ln(A)\rangle) = \alpha$, the logarithmic gain of fluctuations in A (extrinsic noise sources) transmitted to X, which we assume is

independent of the value of B and of the size of the fluctuation. Selecting $\alpha = 1$ results in the same noise breakdown as Elowitz et al.[7], where the extrinsic noise is the normalized covariance of both reporters, and the intrinsic noise is the total CV-squared minus the extrinsic noise.

Intuitively, the inverse tangent of α is the slope of the data on a log-log plot that lies along the 45-degree diagonal in the special case $\alpha = 1$ but does not if the two reporters experience different fluctuation magnitudes from extrinsic sources due to the presence of noise-changing regulatory components.

We take the logarithm to convert to a linear model to find the components.

$$\log(\mathrm{X}) = \alpha \log(\mathrm{A}) + \log(\mathrm{B})$$

$$\log(\mathrm{X}) = \log(\mathrm{A}) + \log(\mathrm{C})$$

Once more dropping the log notation for simplicity,

$$\mathrm{X} = \alpha\mathrm{A} + \mathrm{B}$$

$$\mathrm{Y} = \mathrm{A} + \mathrm{C}$$

Taking the covariance of the logarithms of the reporters (this can be done directly with cytometry data – gate as in figure S1, take the log of the raw reporter values, and calculate the covariance):

$$\mathrm{Cov}(\mathrm{X}, \mathrm{Y}) = \mathrm{Cov}(\alpha\mathrm{A} + \mathrm{B}, \mathrm{A} + \mathrm{C})$$

$$= \mathrm{Cov}(\alpha\mathrm{A}, \mathrm{A}) + \mathrm{Cov}(\mathrm{B}, \mathrm{A}) + \mathrm{Cov}(\alpha\mathrm{A}, \mathrm{C}) + \mathrm{Cov}(\mathrm{B}, \mathrm{C})$$

Because A, B, and C are defined as uncorrelated,

$$\mathrm{Cov}(\mathrm{X}, \mathrm{Y}) = \mathrm{Cov}(\alpha\mathrm{A}, \mathrm{A}) = \alpha\,\mathrm{Var}(\mathrm{A})$$

We take the variances of the logarithms of X and Y, which are the experimentally determined total noises,

$$\mathrm{Var}(\mathrm{X}) = \alpha^2\mathrm{Var}(\mathrm{A}) + \mathrm{Var}(\mathrm{B})$$

$$\mathrm{Var}(Y) = \mathrm{Var}(A) + \mathrm{Var}(C)$$

Next, we replace the variance of A with the experimental covariance term,

$$\mathrm{Var}(X) = \alpha\,\mathrm{Cov}(X, Y) + \mathrm{Var}(B)$$

$$\mathrm{Var}(Y) = \mathrm{Cov}(X, Y)/\alpha + \mathrm{Var}(C) \qquad \text{(I)}$$

Our goal is to compute α_i (where $i = 1, 2, \dots, N$ ranges over possible inducer concentrations), $\mathrm{Var}(A_i)$, $\mathrm{Var}(B_i)$, and $\mathrm{Var}(C_i)$, from the knowledge of $\mathrm{Cov}(X_i, Y_i)$, $\mathrm{Var}(X_i)$, and $\mathrm{Var}(Y_i)$ Here, the covariance terms correspond to the effects of the extrinsic noise, and the B and C terms are intrinsic noises. So far we have 2N equations (the expressions for $\mathrm{Var}(X_i)$ and $\mathrm{Var}(Y_i)$) in 3N unknowns (α_i and the variances of B and C for each well), but we also know that the inducer of the regulated protein (in our case IPTG) has no effect on the global fluctuations that contribute to noise in the constitutive reporter. This means the extrinsic noise term in (I), $\mathrm{Cov}(X_i, Y_i)/\alpha_i$, (and directly $\mathrm{Var}(Y_i)$ and $\mathrm{Var}(C_i)$ also) should be the same for all inducer conditions, that is, we have $N - 1$ additional equations

$$\frac{\mathrm{Cov}(X_i, Y_i)}{\alpha_i} = \frac{\mathrm{Cov}\left(X_j, Y_j\right)}{\alpha_j} \qquad \text{(II)}$$

for all i, j. Finally, $\alpha_N = 1$ for the unregulated control experiment (in our case fully induced with IPTG), so both constitutively produced reporters have the same extrinsic noise, as in Elowitz et al [7].

The choice of $\alpha_N = 1$ sets the noise components for the fully induced well, but we need to calculate α_i for the other wells. For wells without full IPTG induction, it follows from (II) that we can set α_i equal to $\frac{\mathrm{Cov}(X_i, Y_i)\,\alpha_N}{\mathrm{Cov}(X_N, Y_N)}$, i.e. the ratio of the covariance of the current well to the extrinsic noise of the unregulated control experiment. This forces the extrinsic noise of the constitutive reporter ($\mathrm{Cov}(X_i, Y_i)/\alpha_i$) to the same value $\mathrm{Cov}(X_N, Y_N)/\alpha_N$ for all wells. The computed α_i are then used to calculate the

extrinsic noise of the regulated protein $\alpha_i \mathrm{Cov}(X_i, Y_i)$ for each well, and by subtraction we then obtain the intrinsic components.

All of these terms $\mathrm{Cov}(X_i, Y_i)$ are unbiased estimates of the sample covariance computed as follows, for cytometry data where each individual well i has m cells recorded with reporter measurements x_i^j and y_i^j indexed $j = 1, 2, \ldots, m$:

$$\mathrm{Cov}(\log(X_i), \log(Y_i)) = \frac{1}{m-1}\sum_{j=1}^{m}\left[\left(\log(x_i^j) - \frac{1}{m}\sum\nolimits_{k=1}^{m}\log(x_i^k)\right)\left(\log(y_i^j) - \frac{1}{m}\sum\nolimits_{k=1}^{m}\log(y_i^k)\right)\right]$$

We note that in some experiments the two reporters in the fully induced well do not have the identical statistics required by the dual reporter theory of Elowitz et al. We attribute this to the difficulty in finding statistically identical genes and promoters, and also to measurement related issues. For these experiments, instead of assuming the both reporters have the same intrinsic and extrinsic noise, we may assume that they are proportionally the same. For example, if one reporter's CV-square is 1.2 times the other's we can assume its intrinsic and extrinsic CV-squares are also 1.2 times as large. Hence for the computation, instead of assuming $\alpha_N = 1$ for the fully induced well, we would assume α_N is the ratio of the CVs of the reporters. Otherwise the calculation proceeds the same way.

In summary, this is how we define the noise components, using as data ${n_{totX}}^2$ and ${n_{totY}}^2$, the experimentally determined CV-squares of reporters X and Y (which, as described below, we approximate with the variances of the logs of X and Y) and the covariance of the logarithms of X and Y, also determined experimentally (inducer concentrations are indexed $i = 1, 2, \ldots, N$, where well N is fully induced):

$$\alpha_N = \frac{n_{totX}}{n_{totY}}$$

$$\alpha_i = \frac{\mathrm{Cov}(X_i, Y_i)\,\alpha_N}{\mathrm{Cov}(X_N, Y_N)}, i \neq N$$

$$n_{extX}{}^2 = \alpha_i \, \mathrm{Cov}(X_i, Y_i)$$

$$n_{intX}{}^2 = n_{totX}{}^2 - n_{extX}{}^2$$

$$n_{extY}{}^2 = \mathrm{Cov}(X_i, Y_i)/\alpha_i$$

$$n_{intY}{}^2 = n_{totY}{}^2 - n_{extY}{}^2$$

As mentioned earlier, we have calculated the variances of the logarithms of the reporters instead of the CV-squared. This argument relies on a linearized approximation: the standard deviations of the log-scale variables are approximately the relative standard deviations of the original variables. Indeed, for small values of $\log(X) - \mu_{\log(X)}$:

$$E\left(\left(\log(X) - \mu_{\log(X)}\right)^2\right) \cong E\left(\left(\frac{X - \mu_X}{\mu_X}\right)^2\right) = \frac{E((X - \mu_X)^2)}{\mu_X{}^2}$$

Thus, our computed quantities are approximations of the squared coefficient of variation, which is a standard measure of noise[7]. Note that this strategy replaces the data normalization performed in Elowitz et al. but performs a similar function. This approximation is very close for tight distributions, but gets worse for broad distributions (i.e., the approximation is worse for larger values of $\log(X) - \mu_{\log(X)}$).

We can verify the approximation by calculating the standard deviation of the logarithm of the data and comparing it to the RSD of the original data. We improve the approximation by trimming the largest values of $\log(X) - \mu_{\log(X)}$ by dropping all values more than 2.5 standard deviations from the mean of the log of the data (these points are not dropped from the direct RSD verification, and the cutoff was obtained by trying several values).

Verification and decomposition of simulated noise

In our noise decomposition, we expect random quantities which affect the expression of both genes to show up as extrinsic noise, while we expect random quantities which affect only a single gene to show up

as intrinsic. We address the case where one reporter may be less sensitive to extrinsic noise sources due to noise-reducing regulatory pathways. To see this, first take the simplest case of a two-color experiment: suppose we have a plasmid with a constitutive bidirectional promoter P coding for reporters X and Y, and let the only source of uncertainty be the plasmid copy number. Then we have production rates of each reporter:

$$\frac{dX}{dt} = k_1 P - k_2 X$$

$$\frac{dY}{dt} = k_3 P - k_4 Y$$

At steady-state, we have the relations

$$X = \frac{k_1}{k_2} P \rightarrow \log(X) = \log\left(\frac{k_1}{k_2}\right) + \log(P)$$

$$Y = \frac{k_3}{k_4} P \rightarrow \log(Y) = \log\left(\frac{k_3}{k_4}\right) + \log(P)$$

And we want to find the extrinsic noise, the normalized covariance, which we have defined approximately by taking the covariance of the logarithm of the data:

$$n_{ext}^2 = \mathrm{Cov}(\log(X), \log(Y))$$

$$= \mathrm{Cov}\left(\log\left(\frac{k_1}{k_2}\right) + \log(P), \log\left(\frac{k_3}{k_4}\right) + \log(P)\right)$$

$$= \mathrm{Cov}\left(\log\left(\frac{k_1}{k_2}\right), \log\left(\frac{k_3}{k_4}\right)\right) + \mathrm{Cov}\left(\log\left(\frac{k_1}{k_2}\right), \log(P)\right) + \mathrm{Cov}\left(\log(P), \log\left(\frac{k_3}{k_4}\right)\right)$$
$$+ \mathrm{Cov}(\log(P), \log(P))$$

$$n_{ext}^2 = \mathrm{Cov}(\log(P), \log(P)) = \mathrm{Var}(\log(P))$$

To calculate intrinsic noise we need the total noise of each reporter:

$$n_{totX}{}^2 = Var(log(X)) = Var\left(log\left(\frac{k_1}{k_2}\right) + log(P)\right) = Var(log(P))$$

$$n_{totY}{}^2 = Var(log(Y)) = Var\left(log\left(\frac{k_3}{k_4}\right) + log(P)\right) = Var(log(P))$$

Which shows that in this example there is no intrinsic noise; hence a common promoter for two reporters is an extrinsic source of noise:

$$n_{intX}{}^2 = n_{totX}{}^2 - n_{ext}{}^2 = 0$$

$$n_{intY}{}^2 = n_{totY}{}^2 - n_{ext}{}^2 = 0$$

Suppose instead that there were two different plasmids with promoters P1 and P2 coding for reporters X and Y, and let their copy number be independent random variables. Setting up the problem the same way,

$$\frac{dX}{dt} = k_1P_1 - k_2X$$

$$\frac{dY}{dt} = k_3P_2 - k_4Y$$

At steady-state,

$$X = \frac{k_1}{k_2}P_1 \rightarrow log(X) = log\left(\frac{k_1}{k_2}\right) + log(P_1)$$

$$Y = \frac{k_3}{k_4}P_2 \rightarrow log(Y) = log\left(\frac{k_3}{k_4}\right) + log(P_2)$$

Calculating the extrinsic noise,

$$n_{ext}{}^2 = \ Cov(log(X)\,, log(Y))$$

$$= Cov\left(log\left(\frac{k_1}{k_2}\right) + log(P_1), log\left(\frac{k_3}{k_4}\right) + log(P_2)\right)$$

$$= \mathrm{Cov}\left(\log\left(\frac{k_1}{k_2}\right), \log\left(\frac{k_3}{k_4}\right)\right) + \mathrm{Cov}\left(\log\left(\frac{k_1}{k_2}\right), \log(P_2)\right) + \mathrm{Cov}\left(\log\ (P_1), \log\left(\frac{k_3}{k_4}\right)\right)$$

$$+ \mathrm{Cov}(\log\ (P_1), \log(P_2))$$

$$n_{ext}{}^2 = \mathrm{Cov}(\log\ (P_1), \log(P_2)) = 0$$

For the total noise,

$$n_{totX}{}^2 = \mathrm{Var}(\log(P_1))$$

$$n_{totY}{}^2 = \mathrm{Var}(\log(P_2))$$

$$n_{intX}{}^2 = n_{totX}{}^2 - n_{ext}{}^2 = \mathrm{Var}(\log(P_1))$$

$$n_{intY}{}^2 = n_{totY}{}^2 - n_{ext}{}^2 = \mathrm{Var}(\log(P_2))$$

Hence in this case, where the random variable independently affects the two reporters, the extrinsic noise is zero, making these intrinsic noise sources.

Notice that the strength of our approach is when the two reporters are not identically regulated with identical statistics. We extend the applicability by assigning different extrinsic noise quantities to each reporter, so that now instead of there being a single extrinsic noise, each reporter has its own set of intrinsic and extrinsic contributions. The following example shows what can happen to extrinsic noise in the case of negative feedback. Suppose we have the extrinsic promoter case, but reporter X has negative feedback (and $k_5X \gg 1$):

$$\frac{dX}{dt} = k_1P\frac{1}{1 + k_5X} - k_2X \approx \frac{k_1P}{k_5X} - k_2X$$

$$\frac{dY}{dt} = k_3P - k_4Y$$

At steady-state,

$$X^2 = \frac{k_1 P}{k_2 k_5} \rightarrow \log(X^2) = \log\left(\frac{k_1}{k_2 k_5}\right) + \log(P) \rightarrow \log(X) = k + \frac{1}{2}\log(P)$$

$$Y = \frac{k_3}{k_4} P \rightarrow \log(Y) = \log\left(\frac{k_3}{k_4}\right) + \log(P)$$

Calculating the extrinsic noise,

$$n_{ext}{}^2 = \text{Cov}(\log(X), \log(Y))$$

$$= \text{Cov}\left(k + \frac{1}{2}\log(P), \log\left(\frac{k_3}{k_4}\right) + \log(P)\right)$$

$$= \text{Cov}\left(k, \log\left(\frac{k_3}{k_4}\right)\right) + \text{Cov}(k, \log(P)) + \text{Cov}\left(\log\ (P), \log\left(\frac{k_3}{k_4}\right)\right) + \text{Cov}\left(\frac{1}{2}\log\ (P), \log(P)\right)$$

$$n_{ext}{}^2 = \text{Cov}\left(\frac{1}{2}\log\ (P), \log(P)\right) = \frac{1}{2}\text{Var}(\log(P))$$

For the total noise,

$$n_{totX}{}^2 = \text{Var}(\log(X)) = \text{Var}\left(k + \frac{1}{2}\log(P)\right) = \frac{1}{4}\text{Var}(\log(P))$$

$$n_{totY}{}^2 = \text{Var}(\log(Y)) = \text{Var}\left(\log\left(\frac{k_3}{k_4}\right) + \log(P)\right) = \text{Var}(\log(P))$$

If we calculate the intrinsic noise using the Elowitz et al. approach, we find that the extrinsic noise exceeds the total noise for reporter X. However, for this simplified example, we know the only noise source is an extrinsic variable, and thus the intrinsic noise should turn out to be zero. This allows us to infer that α, as described in the previous section, has a value of ½, representing the fact that reporter X experiences half as much noise from the variable plasmid copy number as Y does.

$$n_{extX}{}^2 = \frac{\alpha}{2} Var(log(P)) = \frac{1}{4} Var(log(P))$$

$$n_{extY}{}^2 = \frac{1}{2\alpha} Var(log(P)) = Var(log(P))$$

$$n_{intX}{}^2 = n_{intY}{}^2 = 0$$

Recall that in the experiments, we must first estimate α in a case where the Elowitz et. al. assumptions hold, i.e., the reporters are identically regulated.

To confirm our analysis we used simulations to test the decomposition on noise for two extreme cases, where we control the levels of intrinsic and extrinsic noise. As illustrated in **Supplementary Fig. 13a** we first we vary the strength of transcription of a single bidirectional promoter coding for two fluorescent proteins, leading to perfectly correlated fluorescence quantities, which our decomposition shows to have only extrinsic noise and no intrinsic noise. Next, in **Supplementary Fig. 13b** we vary the strength of transcription of two fluorescent genes independently, which leads to uncorrelated fluorescence quantities; our method returns only intrinsic noise and no extrinsic noise.

Furthermore, in **Supplementary Fig. 14,** we simulated mixtures of both noise types, generated by varying the strength of transcription of two different reporter genes as in the intrinsic noise case in the previous figure, but also by varying the amount of a transcription factor which regulates both genes. For panel a, the noise breakdown gives an intrinsic noise of 0.22 and an extrinsic noise of 0.16 for both proteins. For panel b, where we give the common transcription factor extra variability, it raises the extrinsic noise substantially (but not the intrinsic noise; the common transcription factor is an "extrinsic variable"). The intrinsic noise changes slightly to 0.21 and the extrinsic noise jumps to 0.23.

References

1. Hoebeeck, J., Speleman, F. & Vandesompele, J. in (eds Hilario, E. & Mackay, J.) 205-226 (Humana Press, 2007).

2. Bodin, L., Beaune, P. H. & Loriot, M. Determination of Cytochrome P450 2D6 (CYP2D6) Gene Copy Number by Real-Time Quantitative PCR. *Journal of Biomedicine and Biotechnology* **2005**, 248-253 (2005).

3. Zheng, S. *et al*. DNA hypermethylation profiles associated with glioma subtypes and EZH2 and IGFBP2 mRNA expression. *Neuro-oncology* **13**, 280-289 (2011).

4. Li, Y., Moore, R., Guinn, M. & Bleris, L. Transcription activator-like effector hybrids for conditional control and rewiring of chromosomal transgene expression. *Scientific Reports* **2:897** (2012).

5. Swain, P. S., Elowitz, M. B. & Siggia, E. D. Intrinsic and extrinsic contributions to stochasticity in gene expression. *Proceedings of the National Academy of Sciences* **99**, 12795-12800 (2002).

6. Volfson, D. *et al*. Origins of extrinsic variability in eukaryotic gene expression. *Nature* **439**, 861-864 (2006).

7. Elowitz, M., Levine, A., Siggia, E. & Swain, P. Stochastic Gene Expression in a Single Cell. *Science* **297**, 1183-1186 (2002).

8. Bar-Even, A. *et al*. Noise in protein expression scales with natural protein abundance. *Nature Genetics* **38**, 636-643 (2006).

9. Newman, J. R. S. *et al*. Single-cell proteomic analysis of S. cerevisiae reveals the architecture of biological noise. *Nature* **441**, 840-846 (2006).

10. Paulsson, J. Summing up the noise in gene networks. *Nature* **427**, 415-418 (2004).
